# Heterogeneous ensembles for predicting survival of metastatic, castrate-resistant prostate cancer patients

**DOI:** 10.12688/f1000research.8231.3

**Published:** 2017-07-06

**Authors:** Sebastian Pölsterl, Pankaj Gupta, Lichao Wang, Sailesh Conjeti, Amin Katouzian, Nassir Navab

**Affiliations:** 1 Computer Aided Medical Procedures, Technical University of Munich, Munich, Germany; 2 Johns Hopkins University, Baltimore, USA

**Keywords:** survival analysis, censoring, prostate cancer, ensemble learning, heterogeneous ensemble

## Abstract

Ensemble methods have been successfully applied in a wide range of scenarios, including survival analysis. However, most ensemble models for survival analysis consist of models that all optimize the same loss function and do not fully utilize the diversity in available models. We propose heterogeneous survival ensembles that combine several survival models, each optimizing a different loss during training. We evaluated our proposed technique in the context of the Prostate Cancer DREAM Challenge, where the objective was to predict survival of patients with metastatic, castrate-resistant prostate cancer from patient records of four phase III clinical trials. Results demonstrate that a diverse set of survival models were preferred over a single model and that our heterogeneous ensemble of survival models outperformed all competing methods with respect to predicting the exact time of death in the Prostate Cancer DREAM Challenge.

## Introduction

Today, Cox’s proportional hazards model ^1^ is the most popular survival model because of its strong theoretical foundation. However, it only accounts for linear effects of the features and is not applicable to data with multicolinearities or high-dimensional feature vectors. In addition to Cox’s proportional hazards model, many alternative survival models exist: accelerated failure time model, random survival forest ^2^, gradient boosting ^3, 4^, or support vector machine ^5– 9^. Often it is difficult to choose the best survival model, because each model has its own advantages and disadvantages, which requires extensive knowledge of each model. Ensembles techniques leverage multiple decorrelated models – called base learners – by aggregating their predictions, which often provides an improvement over a single base learner if base learners’ predictions are *accurate* and *diverse* ^10, 11^. The first requirement states that a base learner must be better than random guessing and the second requirement states that predictions of any two base learners must be uncorrelated. The base learners in most ensemble methods for survival analysis are of the same type, such as survival trees in a random survival forest ^2^.

Caruana **et al**. ^12^ proposed *heterogeneous ensembles* for classification, where base learners are selected from a library of many different types of learning algorithms: support vector machines, decision trees, *k* nearest neighbor classifiers, and so forth. In particular, the library itself can contain other (homogeneous) ensemble models such that the overall model is an ensemble of ensembles. The ensemble is constructed by estimating the performance of models in the library from a separate validation set and iteratively selecting the model that increases ensemble performance the most, thus satisfying the first requirement with respect to the accuracy of base learners. To ensure that models are diverse, which is the second requirement, Margineant and Dietterich ^13^ proposed to use Cohen’s kappa ^14^ to estimate the degree of disagreement between any pair of classifiers. The *S* pairs with the lowest kappa statistic formed the final ensemble. In addition, Rooney *et al*. ^15^ proposed a method to construct a heterogeneous ensemble of regression models by ensuring that residuals on a validation set are uncorrelated.

We present heterogeneous survival ensembles to build an ensemble from a wide range of survival models. The main advantage of this approach is that it is not necessary to rely on a single survival model and any assumptions or limitations that model may imply. Although predictions are real-valued, a per-sample error measurement, similar to residuals in regression, generally does not exist. Instead, the prediction of a survival model consists of a risk score of arbitrary scale and a direct comparison of these values, e.g., by computing the squared error, is not meaningful. Therefore, we propose an algorithm for pruning an ensemble of survival models based on the correlation between predicted risk scores on an independent test set. We demonstrate the advantage of heterogeneous survival ensembles in the context of the Prostate Cancer DREAM Challenge ^16^, which asked participants to build a prognostic model to predict overall survival of patients with metastatic, castrate-resistant prostate cancer (mCRPC).

In the early stages of therapy, prostate cancer patients are usually treated with androgen deprivation therapy, but for 10–20% of patients the cancer will inevitably progress from castrate-sensitive to castrate-resistant within 5 years ^17^. The median survival time for patients with mCRPC is typically less than 2 years ^17^. To improve our understanding of mCRPC, the Prostate Cancer DREAM Challenge exposed the community to a large and curated set of patient records and asked participants to 1) predict patients’ overall survival, and 2) predict treatment discontinuation due to adverse events. In this paper, we focus on the first sub challenge, i.e., the prediction of survival. To the best of our knowledge, this is the first scientific work that uses heterogeneous ensembles for survival analysis.

The paper is organized as follows. In the methods section, we briefly describe the framework of heterogeneous ensembles proposed by Caruana *et al*. ^12^ and Rooney *et al*. ^15^ and propose an extension to construct a heterogeneous ensemble of survival models. Next, we present results of three experiments on data of the Prostate Cancer DREAM Challenge, including our final submission under the name Team CAMP. Finally, we discuss our results and close with concluding remarks.

## Methods

Caruana *et al*. ^12^ formulated four basic steps to construct a heterogeneous ensemble:

1. Initialize an empty ensemble.
2. Update the ensemble by adding a model from the library that maximizes the (extended) ensemble’s performance on an independent validation (hillclimb) set.
3. Repeat step 2 until the desired size of the ensemble is reached or all models in the library have been added to the ensemble.
4. Prune the ensemble by reducing it to the subset of base learners that together maximize the performance on a validation (hillclimb) set.

By populating the library with a wide range of algorithms, the requirement of having a diverse set of base learners is trivially satisfied. In addition, each model can be trained on a separate bootstrap sample of the training data. The second step ensures that only accurate base learners are added to the ensemble, and the fourth step is necessary to avoid overfitting on the validation set and to ensure that the ensemble comprises a diverse group of base learners. These two steps are referred to as *ensemble selection* and *ensemble pruning* and are explained in more detail below.

### Efficient ensemble selection

The algorithm by Caruana *et al*. ^12^ has the advantage that models in the library can be evaluated with respect to any performance measure. The final heterogeneous ensemble maximizes the selected performance measure by iteratively choosing the best model from the library. Therefore, the training data ** needs to be split into two non-overlapping parts: one part ( **_train_) used to train base learners from the library, and the other part ( **_val_) used as the validation set to estimate model performances. Data in the biomedical domain is usually characterized by small sample sizes, which would lead to an even smaller training set if a separate validation set is used. Caruana *et al*. ^18^ observed that if the validation set is small, the ensemble tends to overfit more easily, which is especially concerning when the library contains many models. To remedy this problem, Caruana *et al*. [ 18, p. 3] proposed a solution that “embed[ded] cross-validation within ensemble selection so that all of the training data can be used for the critical ensemble hillclimbing step.” Instead of setting aside a separate validation set, they proposed to use *cross-validated models* to determine the performance of models in the library (see Algorithm 1).

**Algorithm 1. Ensemble selection for survival analysis**

    
**Input**: Library of *N* base survival models, training data ,

                number of folds *K*, minimum desired performance *c*_min_.

    
**Output**: Ensemble of base survival models exceeding

                   minimum performance.

1   ← ∅

2 **for** *i* ← 1 **to** *N* **do**

3 *_i_* ← ∅

4 **for** *k* ← 1 **to** *K* **do**

5 *^k^*_train_ ← *k*-th training set

6 *^k^*_test_ ← *k*-th test set

7 *M _ik_* ← Train *k*-th sibling of *i*-th survival model on *^k^*_train_

8 *c _k_* ← Prediction of survival model *M _ik_* on *^k^*_test_

9 *_i_* ← *_i_* ∪ {( *^k^*_test_, *c _k_*)} /* Store prediction and

               
associated ground truth */

10 **end**

11 *c̄ _i_* ← Performance of *i*-th survival model based on

          predictions and ground truth in *_i_*

12 **if** *c̄ _i_* ≥ *c*_min_ **then**

13 ** ← ** ∪ {( *M _i_*_1_, …, *M _iK_* , *c̄ _i_*)} /* Store *K*

                  
siblings and performance of *i*-th model */

14 **end**

15 **end**

16 **return** Base models in **

A cross-validated model is itself an ensemble of identical models, termed *siblings*, each trained on a different subset of the training data. It is constructed by splitting the training data into *K* equally sized folds and training one identically parametrized model on data from each of the *K* combinations of *K −* 1 folds. Together, the resulting *K* siblings form a cross-validated model.

To estimate the performance of a cross-validated model, the complete training data can be used, because the prediction of a sample *i* in the training data ** only comes from the sibling that did not see that particular sample during training, i.e., for which *i* ∉ **_train_. Therefore, estimating the performance using cross-validated models has the same properties as if one would use a separate validation set, but without reducing the size of the ensemble training data. If a truly new data point is to be predicted, the prediction of a cross-validated model is the average of the predictions of its siblings. Algorithm 1 summarizes the steps in building a heterogeneous ensemble from cross-validated survival models.

Note that if a cross-validated survival model is added to the ensemble, the ensemble actually grows by *K* identically parametrized models of the same type – the siblings (see line 13 in Algorithm 1). Therefore, the prediction of an ensemble consisting of *S* cross-validated models is in fact an ensemble of *K × S* models.

### Ensemble pruning

Ensemble selection only ensures that base learners are better than random guessing, but does not guarantee that predictions of base learners are diverse, which is the second important requirement for ensemble methods ^10, 11^.

In survival analysis, predictions are real-valued, because they either correspond to a risk score or to the time of an event. Therefore, we adapted a method for pruning an ensemble of regression models that accounts for a base learner’s accuracy and correlation to other base learners ^15^, as illustrated below.

**Pruning regression ensembles.** Given a library of base learners, first, the performance of each base learner is estimated either from a separate validation set or via cross-validated models following Algorithm 1. To estimate the diversity of a pair of regression models, Rooney *et al*. ^15^ considered a model’s residuals as a per-sample error measurement. Given the residuals of two models on the same data, it is straightforward to obtain a measure of diversity by computing Pearson’s correlation coefficient. They defined the diversity of a single model based on the correlation of its residuals to the residuals of all other models in the ensemble and by counting how many correlation coefficients exceeded a user-supplied threshold *τ*_corr_. The diversity score can be computed by subtracting the number of correlated models from the total number of models in the ensemble and normalizing it by the ensemble size. If a model is sufficiently correlated with all other models, its diversity is zero, while if it is completely uncorrelated, its diversity is one. Moreover, they defined the accuracy of the *i*-th model relative to the root mean squared error (RMSE) of the best performing model as accuracy( *i*) = (min _*j*=1,..., *S*_ RMSE( *j*)) */*RMSE( *i*). Finally, Rooney *et al*. ^15^ added the diversity score of each model to its accuracy score and selected the top *S* base learners according to the combined accuracy-diversity score. Algorithm 2 summarizes the algorithm by Rooney *et al*. ^15^, where the correlation function would compute Pearson’s correlation coefficient between residuals of the *i*-th and *j*-th model.

**Algorithm 2. Ensemble pruning algorithm of Rooney *et al.*^15^**

    
**Input**: Set of base survival models ** and their average

                cross-validation performance, validation set **_val_,

                desired size *S* of ensemble, correlation threshold τ _corr_.

    
**Output**: Aggregated predictions of *S* base survival models.

1 *c*_max_ ← Highest performance score of any model in **

2 **if** | ** | > *S* **then**

3 **  ← ∅

4 **for** *i* ← 1 **to** | ** | **do**

5 *p _i_* ← Prediction of data **_val_ using *i*-th base survival

                model in **

6 *count* ← 0

7 **for** *j* ← 1 **to** | ** | **do**

8 *p _j_* ← Prediction of data **_val_ using *j*-th base

                       survival model in **

 9 **if** *i* ≠ *j* ∧ correlation( *p _i_*, *p _j_*, **_val_) ≥ τ _corr_ **then**

 10 *count* ← *count* + 1

11 **end**

12 **end**

13 *d _i_* ← ( | ** | – *count*)/ | ** |

14 *c̄ _i_* ← Average cross-validation performance of *i*-th

                survival model in

15             ← ∪ {( *i*, *c̄ _i_*/ *c*_max_ + *d _i_*)}

16 **end**

17 *** ← Top *S* survival models with highest score according

             to **

18 **else**

19 *** ← **

20 **end**

21 **return** Prediction of **_val_ by aggregating predictions of base

      learners in survival ensemble ***

**Pruning survival ensembles.** If the library consists of survival models rather than regression models, a persample error, similar to residuals in regression, is difficult to define. Instead, predictions are risk scores of arbitrary scales and the ground truth is the time of an event or the time of censoring. Hence, a direct comparison of a predicted risk score to the observed time of an event or the time of censoring, for instance via the squared error, is not meaningful. We propose to measure the diversity in an ensemble based on the correlation between predicted risk scores, i.e., independent of the ground truth. Here, we consider two correlation measures:

1. Pearson’s correlation coefficient, and
2. Kendall’s rank correlation coefficient (Kendall’s *τ*).

Hence, we measure the diversity of a heterogeneous ensemble of survival models without requiring ground truth or a separate validation set. We believe this is not a disadvantage, because the combined score in line 15 of Algorithm 2 already accounts for model accuracy, which could be estimated by the concordance index ^19^ or integrated area under the time-dependent ROC curve ^20, 21^ on a validation set or using Algorithm 1. In fact, since the diversity score for survival models does not depend on ground truth, the pruning step can be postponed until the prediction phase – under the assumption that prediction is always performed for a set of samples and not a single sample alone. Consequently, the ensemble will not be static anymore and is allowed to change if new test data is provided, resulting in a dynamic ensemble.

In summary, for pruning an ensemble of survival models, Algorithm 2 is applied during prediction with the following modifications:

1. Replace validation data **_val_ by the feature vectors of the test data ****X****_new_.
2. Compute the performance score using the concordance index ^19^, integrated area under the time-dependent, cumulative-dynamic ROC curve ^20, 21^ or any other performance measure for censored outcomes.
3. Measure the correlation among predicted risk scores using Pearson’s correlation coefficient or Kendall’s rank correlation coefficient.

The prediction of the final ensemble is the average predicted risk score of all its members after pruning.

## Experiments

### Data

**Figure 1.**
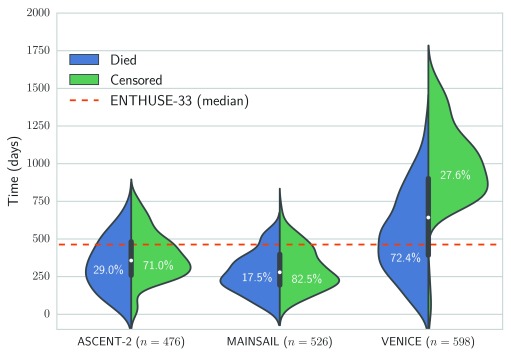
Overview of distribution of survival and censoring times in training data from the ASCENT-2, VENICE, MAINSAIL, and ENTHUSE-33 trial ^22– 25^. Numbers in brackets denote the total number of patients in the respective trial, and the dashed line is the median follow-up time in the ENTHUSE-33 trial, which was used as independent test data.

The Prostate Cancer DREAM Challenge ^16^ provided access to 1,600 health records from three separate phase III clinical trials for training ^22– 24^, and data from an independent clinical trial of 470 men for testing (values of dependent variables were held back and not revealed to participants) ^25^. Figure 1 illustrates the distribution of censoring and survival times of the respective trials. The median follow-up time for the MAINSAIL trial ^23^, the ASCENT-2 trial ^22^, and VENICE trial ^24^ was 279, 357, and 642.5 days, respectively. For the test data from the ENTHUSE-33 trial ^25^, the median follow-up was 463 days.

We partitioned the training data into 7 sets by considering all possible combinations of the three trials constituting the training data (see Table 1). Each partition was characterized by a different set of features, ranging between 383 features for data from the MAINSAIL trial to 217 features when combining data of all three trials. Features were derived from recorded information with respect to medications, comorbidities, laboratory measurements, tumor measurements, and vital signs (see supplementary material for details). Finally, we used a random survival forest ^2^ to impute missing values in the data.

**Table 1.** Different sets of features that were constructed by considering the intersection between trials in the Prostate Cancer DREAM Challenge.

ASCENT-2	MAINSAIL	VENICE	Samples	Features
•	•	•	1,600	217
	•	•	1,124	345
•		•	1,074	220
•	•		1,002	221
		•	598	350
	•		526	383
•			476	223

### Validation scheme

We performed a total of three experiments, two based on cross-validation using the challenge training data, and one using the challenge test data from the ENTHUSE-33 trial as hold-out data. In the first experiment, we randomly split each of the datasets in Table 1 into separate training and test data and performed 5-fold cross-validation. Thus, test and training data comprised different individuals from the same trial(s). We refer to this scenario as with-in trial validation. In the second experiment, referred to as between trials validation, we used data from one trial as hold-out data for testing and data from one or both of the remaining trials for training. This setup resembles the challenge more closely, where test data corresponded to a separate trial too. We only considered features that were part of both the training and test data. In each experiment above, the following six survival models were evaluated:

1. Cox’s proportional hazards model ^1^ with ridge ( *ι*_2_) penalty,
2. Linear survival support vector machine (SSVM) ^9^,
3. SSVM with the clinical kernel ^26^,
4. Gradient boosting of negative log partial likelihood of Cox’s proportional hazards model ^3^ with randomized regression trees as base learners ^27, 28^,
5. Gradient boosting of negative log partial likelihood of Cox’s proportional hazards model ^3^ with componentwise least squares as base learners ^29^,
6. Random survival forest ^2^.

In addition, the training of each survival model was wrapped by grid search optimization to find optimal hyper-parameters. The complete training data was randomly split into 80% for training and 20% for testing to estimate a model’s performance with respect to a particular hyper-parameter configuration. The process was repeated for ten different splits of the training data. Finally, a model was trained on the complete training data using the hyper-parameters that on average performed the best across all ten repetitions. Performance was estimated by Harrell’s concordance index ( *c* index) ^19^. All continuous features were normalized to zero mean and unit variance and nominal and ordinal features were dummy coded.

For the Prostate Cancer DREAM Challenge’s final evaluation, we built a heterogeneous ensemble from a wide range of survival models. In sub challenge 1a, the challenge organizers evaluated submissions based on the integrated area under the time-dependent, cumulative-dynamic ROC curve (iAUC) ^20, 21^ – integrated over time points every 6 months up to 30 months after the first day of treatment – and in sub challenge 1b, based on the root mean squared error (RMSE) with respect to deceased patients in the test data. The performance of submitted models was estimated based on 1,000 bootstrap samples of the ENTHUSE-33 trial data and the Bayes factor to the top performing model and a baseline model by Halabi *et al*. ^30^ (only for sub challenge 1a). The Bayes factor provides an alternative to traditional hypothesis testing, which relies on *p*-values to determine which of two models is preferred (see e.g. 31). According to Jeffreys ^32^, a Bayes factor in the interval [3; 10] indicates moderate evidence that the first model outperformed the second model and strong evidence if the Bayes factor is greater 10, else evidence is insufficient.

## Results

### With-in trial validation

**Figure 2.**
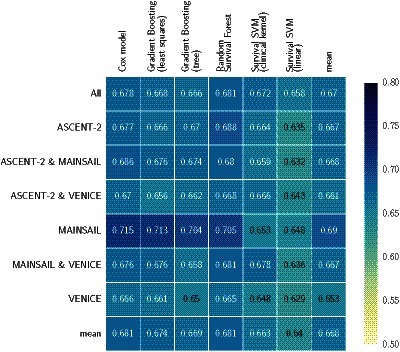
Cross-validation performance of survival models on data from from the ASCENT-2, VENICE, and MAINSAIL trial, as well as any combination of these datasets. The last column (mean) denotes the average performance of all models on a particular dataset and the last row (mean) denotes the average performance of a particular model across all datasets. Numbers indicate the average of Harrell’s concordance index across five cross-validation folds.

Figure 2 summarizes the average cross-validation performance across all five test sets for all seven datasets in Table 1. Overall, the average concordance index ranged between 0.629 and 0.713 with a mean of 0.668. It is noteworthy that all classifiers but SSVM models performed best on data of the MAINSAIL trial, which comprised 526 subjects and the highest number of features among all trials (383 features). A SSVM was likely to have an disadvantage due to the high number of features and because feature selection is not embedded into its training as for the remaining models. In fact, SSVM models performed worst on data from the MAINSAIL and VENICE trials, which were the datasets with the most features. SVM-based models performed best if data from at least two trials were combined, which increased the number of samples and decreased the number of features. Moreover, the results show that linear survival support vector machines performed poorly. A considerable improvement could be achieved when using kernel-based survival support vector machines with the clinical kernel, which is especially useful if data is a mix of continuous, categorical and ordinal features. For low-dimensional data, the kernel SSVM could perform equally well as or better than gradient boosting models, but was always outperformed by a random survival forest.

When considering the performance of models across all datasets (last row in Figure 2), random survival forests and Cox’s proportional hazards models stood out with an average *c* index of 0.681, outperforming the third best: gradient boosting with componentwise least squares base learners. Random survival forests performed better than Cox’s proportional hazards models on 4 out of 7 datasets and was tied on one dataset. The results seem to indicate that a few datasets contain non-linearities, which were captured by random survival forests, but not by gradient boosting with componentwise least squares and Cox’s proportional hazards models. Nevertheless, Cox’s proportional hazards model only performed significantly better than linear SSVM when averaging results over all datasets (see Figure 4).

Finally, we would like to mention that 5 out of 6 survival models performed worst on the VENICE data. Although it contained the largest number of patients, the variance of follow-up times is more than two-fold larger compared to ASCENT-2 and MAINSAIL ( *σ* ^2^ *≈* 342.9 versus 165.1 for ASCENT-2 and 140.2 for MAINSAIL). Moreover, the overlap in the distribution of censoring and survival times was rather small (see Figure 1). Thus, the difference between observed time points in the training and test data based on the VENICE trial is likely more pronounced than for the data from the MAINSAIL or ASCENT-2 trials, which means a survival model has to generalize to a much larger time period. Moreover, the amount of censoring in the VENICE trial is relatively low compared to the other trials. Therefore, the observed drop in performance might stem from the fact that the bias of Harrell’s concordance index usually increases as the amount of censoring increases ^33^. As an alternative, we considered the integrated area under the time-dependent, cumulative-dynamic ROC curve ^20, 21^, which was the main evaluation measure in the Prostate Cancer DREAM Challenge. However, comparing the estimated integrated area under the ROC curve across multiple datasets is not straightforward when follow-up times differ largely among trials (see Figure 1). If the integral is estimated from time points that exceed the follow-up time of almost all patients, the inverse probability of censoring weights used in the estimator of the integrated area under the curve cannot be computed, because the estimated probability of censoring at that time point becomes zero. On the other hand, if time points are defined too conservatively, the follow-up period of most patients will end after the last time point and the estimator would ignore a large portion of the follow-up period. Hence, defining time points that lead to adequate estimates of performance in all three datasets is challenging due to large differences in the duration of follow-up periods.

### Between trials validation

**Figure 3.**
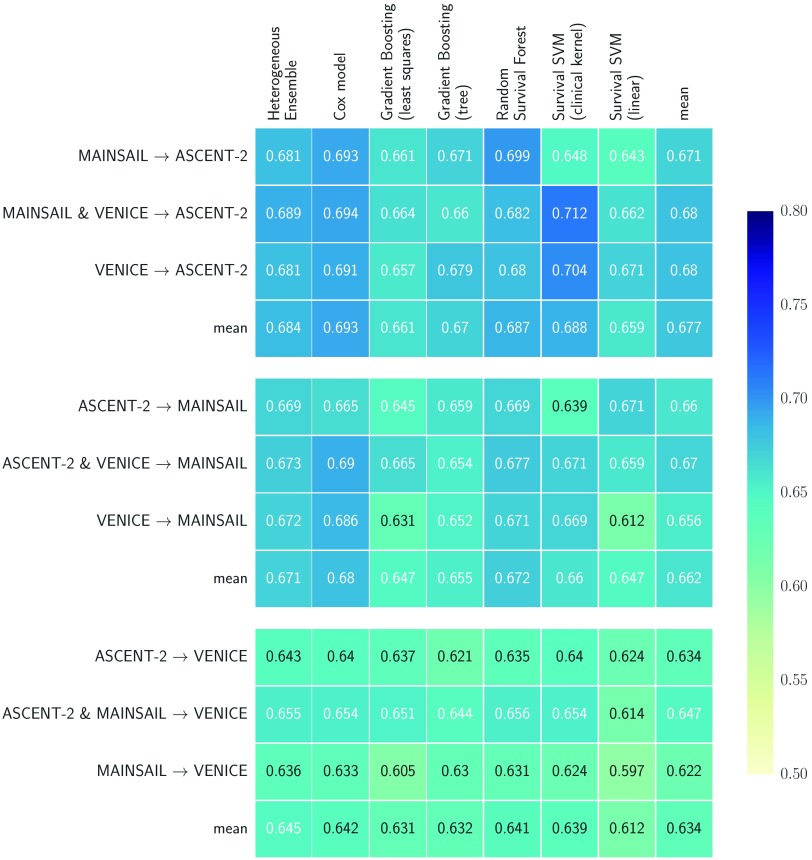
Performance results using hold-out data from from the ASCENT-2, VENICE, and MAINSAIL trial. One trial was used as hold-out data (indicated by the name to the right of the arrow) and one or two of the remaining trials as training data. Numbers indicate Harrell’s concordance index on the hold-out data.

In the second experiment, training and test data were from separate trials, which resembled the setup of the Prostate Cancer DREAM Challenge. We included heterogeneous ensembles in the analyses, trained on a library of models that included multiple copies of each survival model, each with a different hyper-parameter configuration. The library excluded linear SSVM, because it performed poorly in previous experiments, and Cox’s proportional hazards model, because its Newton-Rhapson optimization algorithm used a constant step size instead of a line search, which occasionally led to oscillation around the minimum during ensemble selection. We investigated whether the observed differences in performance are statistically significant by performing a Nemenyi post-hoc test ^34^ based on the results of all train-test-set combinations in Figure 3. Figure 5 summarizes the results.

**Figure 4.**
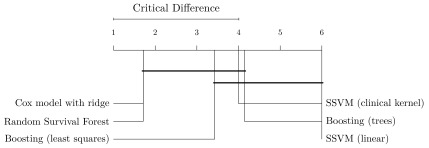
Comparison of methods based on experiments in Figure 2 with the Nemenyi post-hoc test ^34^. Methods are sorted by average rank (left to right) and groups of methods that are not significantly different are connected ( *p*-value >0.05).

**Figure 5.**
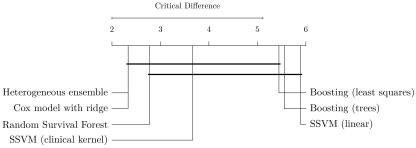
Comparison of methods based on experiments in Figure 3 with the Nemenyi post-hoc test ^34^. Methods are sorted by average rank (left to right) and groups of methods that are not significantly different are connected ( *p*-value >0.05).

The results confirmed observations discussed in the previous section: 1) on average, random survival forests performed better than gradient boosting models and SSVMs, and 2) using SSVM with the clinical kernel was preferred over the linear model. Heterogeneous ensemble ranked first in our experiments, tied with Cox’s proportional hazards model, and significantly outperformed linear SSVM and gradient boosting with regression trees. Among the top five models – which did not perform significantly different from each other – in Figure 5, heterogeneous ensemble stands out by having the lowest variance: its performance ranged between 0.636 and 0.689 (∆ = 0.053), which is a 14% reduction compared to the runner-up (Cox’s model: ∆ = 0.061) and a 12% to 40% reduction when compared to individual base learners in the library (gradient boosting with componentwise least squares: ∆ = 0.060, SSVM with clinical kernel: ∆ = 0.088). The results demonstrate that combining a diverse set of survival models in a heterogeneous ensemble improves performance and increases reliability.

If performance was estimated on the VENICE data, all models performed considerably worse compared to performance estimated on the other datasets. We believe the reason for these results are similar to the cross-validation results on the VENICE data described in the previous section. The bias of Harrell’s concordance index due to vastly different amounts of censoring among trials could be one factor, while the other could be that the follow-up times differed drastically between training and testing. If the follow-up period is much shorter in the training data than in the testing data, it is likely that models generalize badly for time points that were never observed in the training data, which is only the case if the VENICE data is used for testing, but not if data from the MAINSAIL or the ASCENT- 2 trial is used (cf. Figure 1). Interestingly, all models, except linear SSVM, performed best when trained on the maximum number of available patient records, which is different from results in the previous section, where models trained on data with more features performed better.

An unexpected result is that Cox’s proportional hazards model was able to outperform many of the machine learning methods, including random survival forest, which is able to implicitly model non-linear relationships that are not considered by Cox’s proportional hazards model. A possible explanation why the Cox model performed on par with more complicated machine learning methods might be the fact the effective sample size reduces if the amount of censoring increases, as kindly pointed out by one referee. If most samples are censored, the effective size of the study decreases proportionally, which in turn makes it more challenging to reliably identify non-linear effects, which would be the strength of the advanced survival models in our experiments. Following Occam’s razor, the results suggest that, in this case, a simple model is preferred.

Results also indicate that models with embedded feature selection (gradient boosting and random survival forest) were not significantly better than models that take into account all features (Cox model and SSVM with the clinical kernel). The fact that models with embedded feature selection, in particular gradient boosting with componentwise least squares base learner, performed poorly might be false positive selected features, i.e., features that are actually not associated with survival. In high dimensions, methods with embedded feature selection often suffer from instability, i.e., the set of selected features can vary widely when repeatedly fitting a model, e.g., when determining optimal hyper-parameters ^35^. This problem seems to be more pronounced when evaluating models on data from a different study. The number of false positive selections could be controlled by performing stability selection ^35^.

### Challenge hold-out data

To summarize, results presented in the previous two sections demonstrate that

1. SSVM should be used in combination with the clinical kernel.
2. Increasing the number of samples is preferred over increasing the number of features, especially if follow-up periods are large.
3. There is no single survival model that is clearly superior to all other survival models.

From these observations, we concluded that employing heterogeneous survival models, trained on all 1,600 patient records in the training data, would be most reliable. We built two ensembles using Algorithm 1 and Algorithms 2: one maximizing Harrell’s concordance index ^19^, and one minimizing the RMSE. The former was constructed from a library of 1,801 survival models for sub challenge 1a ( *K* = 5, *c*_min_ = 0.66, *τ*_corr_ = 0.6, *S* = 90) and the latter from a library of 1,842 regression models for sub challenge 1b ( *K* = 5, *c*_min_ = 0.85, *τ*_corr_ = 0.6, *S* = 92). We submitted predictions based on these two models to the Prostate Cancer DREAM Challenge. The results in the remainder of this section refer to the final evaluation carried out by the challenge organizers.

**Figure 6.**
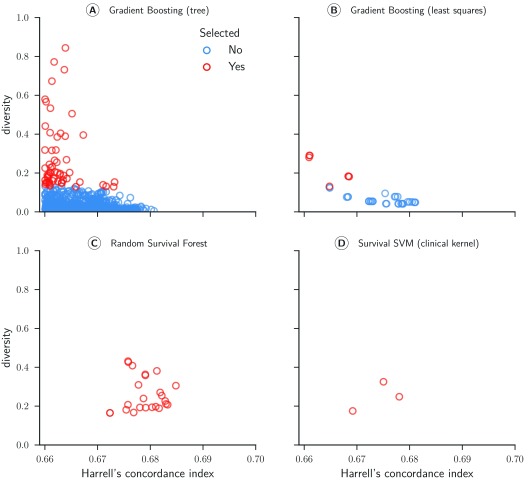
Concordance index and diversity score of 999 survival models for sub challenge 1a. The concordance index was evaluated by cross-validated models on the training data from the from the ASCENT-2, VENICE, and MAINSAIL trial. Diversity was computed based on Pearson’s correlation coefficient between predicted risk scores for 313 patients of the ENTHUSE-33 trial (final scoring set).

**Sub challenge 1a.** Four of the six survival models evaluated in the cross-validation experiments formed the basis of the ensemble (see Table 2). Figure 6 depicts scatter plots comparing models’ performance and diversity. Most of the gradient boosting models with regression trees as base learners were pruned because their predictions were redundant to other models in the ensemble ( Figure 6A). In contrast, all random survival models remained in the ensemble throughout ( Figure 6C). We observed the highest diversity for gradient boosting models (mean = 0.279) and the highest accuracy for random survival forests (mean = 0.679). The final ensemble comprised all types of survival models in the library, strengthening our conclusion that a diverse set of survival models is preferred over a single model.

**Table 2.** Heterogeneous ensemble of survival models used in sub challenge 1a of the Prostate Cancer DREAM Challenge. *All* denotes the initial size of the ensemble, *Pruned* the size after pruning models with Harrell’s concordance index below 0.66, and *Top 5%* to the final size of the ensemble corresponding to the top 5% according the combined accuracy and diversity score in Algorithm 2.

	Configurations		
Survival model	All	Pruned	Top 5%
Gradient boosted Cox model (tree) ^3, 27^	1,728	936	56
Gradient boosted Cox model (least squares) ^3, 29^	36	36	7
Random survival forest ^2^	24	24	24
Ranking-based survival SVM (clinical kernel) ^9, 26^	13	3	3
Σ	1,801	999	90

In the challenge’s final evaluation based on 313 patients of the ENTHUSE-33 trial, 30 out of 51 submitted models outperformed the baseline model by Halabi *et al.* ^30^ by achieving a Bayes factor greater than 3 ^16^. There was a clear winner in team FIMM-UTU and the performance of the remaining models were very close to each other; there was merely a difference of 0.0171 points in integrated area under the ROC curve (iAUC) between ranks 2 and 25 ^16^.

The proposed heterogeneous ensemble of survival models by Team CAMP achieved an iAUC score of 0.7646 on the test data and was ranked 23 ^rd^ according to iAUC and 20 ^th^ according to Bayes factor with respect to the best model (FIMM-UTU). When considering the Bayes factor of the proposed ensemble method to all other models, there is only sufficient evidence (Bayes factor greater 3) that five models performed better (FIMM-UTU, Team Cornfield, TeamX, jls, and KUstat). The Bayes factor to the top two models was 20.3 and 6.6 and ranged between 3 and 4 for the remaining three models. With respect to the model by Halabi *et al*. ^30^, there was strong evidence (Bayes factor 12.2; iAUC 0.7432) that heterogeneous ensembles of survival models could predict survival of mCRPC patients more accurately.

**Sub challenge 1b.** In subchallenge 1b, participants were tasked with predicting the exact time of death rather than ranking patients according to their survival time. Similar to subchallenge 1a, our final model was a heterogeneous ensemble, but based on a different library of models (see Table 3).

**Table 3.** Heterogeneous ensemble used in sub challenge 1b of the Prostate Cancer DREAM Challenge. *All* denotes the initial size of the ensemble, *Pruned* the size after pruning models with a root mean squared error more than 15% above the error of the best performing model, and *Top 5%* to the final size of the ensemble corresponding to the top 5% according the combined accuracy and diversity score in Algorithm 2. AFT: Accelerated Failure Time.

	Configurations		
Regression model	All	Pruned	Top 5%
Gradient boosted AFT model (tree) ^4, 27^	1,728	1,236	90
Gradient boosted AFT model (least squares) ^4, 29^	36	36	0
Hybrid survival SVM (clinical kernel) ^9, 26^	78	9	2
Σ	1,842	1,281	92

**Figure 7.**
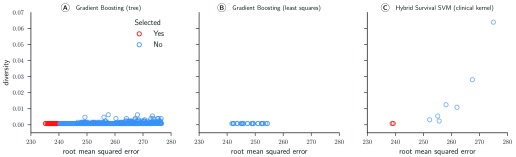
Root mean squared error (RMSE) and diversity score of 1,281 regression models for sub challenge 1b. The RMSE was evaluated by cross-validated models on the training data from the ASCENT-2, VENICE, and MAINSAIL trial. Diversity was computed based on Pearson’s correlation coefficient between residuals on the training data.

Figure 7 illustrates the RMSE and diversity of all 1,281 models after the first pruning step (cf. Table 3). In contrast to the ensemble of survival models used in subchallenge 1a, the ensemble in this subchallenge was characterized by very little diversity: the highest diversity was 0.064. In fact, all 92 models included in the final ensemble had a diversity score below 0.001, which means that pruning was almost exclusively based on the RMSE. Gradient boosting models with componentwise least squares base learners were completely absent from the final ensemble and only two hybrid survival support vector machine models had a sufficiently low RMSE to be among the top 5%.

The evaluation of all submitted models on the challenge’s final test data from the ENTHUSE-33 trial revealed that our proposed heterogeneous ensemble of regression models achieved the lowest root mean squared error (194.4) among all submissions ^16^. The difference in RMSE between the 1 ^st^ placed model and the 25 ^th^ placed model was less than 25. With respect to our proposed winning model, there was insufficient evidence to state it outperformed all other models, because the comparison to five other models yielded a Bayes factor less than three (Team Cornfield, M S, JayHawks, Bmore Dream Team, and A Bavarian dream).

## Discussion

From experiments on the challenge training data, we concluded that it would be best to combine data from all three clinical trials to train a heterogeneous ensemble, because maximizing the number of distinct time points was preferred. Interestingly, the winning team of sub challenge 1a completely excluded data from the ASCENT-2 trial in their solution. They argued that it was too dissimilar to data of the remaining three trials, including the test data ^36^. Therefore, it would be interesting to investigate unsupervised approaches that could deduce a similarity or distance measure between patients, which can be used to decrease the influence of outlying patients during training.

The second important conclusion from our experiments is that no survival model clearly outperformed all other models in all the evaluated scenarios. Our statistical analysis based on results of the between trials validation revealed that Cox’s proportional hazards model performed significantly better than the linear survival support vector machine and gradient boosting with regression trees as base learners, and that the random survival forest performed significantly better than linear survival support vector machines; the remaining differences were deemed statistically insignificant. Therefore, we constructed a heterogeneous ensemble of several survival models with different hyper-parameter configurations and thereby avoided relying only on a single survival model with a single hyper-parameter configuration. In total, we considered two libraries, each consisting of over 1,800 different models, which were pruned to ensure accuracy and diversity of models – we observed only minor differences when substituting Pearson’s correlation for Kendall’s rank correlation during ensemble pruning.

The proposed ensemble approach was able to outperform all competing models in sub challenge 1b, where the task was to predict the exact time of death. In sub challenge 1a, participants had to provide a relative risk score and our ensemble approach was significantly outperformed by five competing models ^16^. Due to large differences in teams’ overall solutions it is difficult to pinpoint the reason for the observed performance difference: it could be attributed to the choice of base learners, or to choices made during pre-processing or filtering the data. From our experience of the three intermediate scoring rounds before the final submission, we would argue that identifying the correct subset of patients in the training data that is most similar to the test data is more important than choosing a predictive model. By training a survival model on data combined from three trials and applying it to patients from a fourth trial, inconsistencies between trials inevitably lead to outliers with respect to the test data, which in turn diminishes the performance of a model – if not addressed explicitly during training.

A possible explanation why the heterogeneous ensemble worked better for survival time prediction (sub challenge 1b) than for risk score prediction (sub challenge 1a) might be that we maximized the concordance index during ensemble construction and not the area under the time-dependent ROC curve, which was used in the challenge’s final evaluation. In addition, we aggregated predictions of survival models by averaging, although predictions of survival models are not necessarily on the same scale. In regression, the prediction is a continuous value that directly corresponds to the time of death, which allows simple averaging of individual predictions. In survival analysis, semantics are slightly different. Although predictions are real-valued as well, the prediction of a survival model does generally not correspond to the time of death, but is a risk score on an arbitrary scale. A homogeneous ensemble only consists of models of the same type, therefore predictions can be aggregated by simply computing the average. A problem arises for heterogeneous ensembles if the scale of predicted risk scores differs among models. To illustrate the problem, consider an ensemble consisting of survival trees as used in a random survival forest ^2^ and ranking-based linear survival support vector machines ^9^. The prediction of the former is based on the cumulative hazard function estimated from samples residing in the leaf node a new sample was assigned to. Thus, predictions are always positive due to the definition of the cumulative hazard function (see e.g. 37). In contrast, the prediction of a linear SSVM is the inner product between a model’s vector of coefficients and a sample’s feature vector, which can take on negative as well as positive values. It is easy to see that, depending on the scale difference, simply averaging predicted risk scores favors models with generally larger risk scores (in terms of absolute value) or positive and negative predicted risk scores cancel each other out. Instead of simply averaging risk scores, the problem could be alleviated if model risk scores were first transformed into ranks, thereby putting them on a common scale, before averaging the resulting ranks. We evaluated this approach after the Prostate Cancer DREAM Challenge ended: averaging ranks instead of raw predicted risk scores increased the iAUC value from 0.7644 to 0.7705 on a random sub sample of the ENTHUSE-33 trial.

Finally, we want to pay particular attention to the challenge of combining multiple patients populations for risk prediction. As mentioned above, the follow-up periods and the information collected for the four studies considered here differed vastly. Figure 5 illustrates that there is no single model equally suitable for all cohorts. This problem arises if prediction models are badly calibrated with respect to the target cohort. If outcome information for the target cohort is available, recalibration methods can be used to improve calibration and discrimination of the risk score ^38– 41^. In the context of the Prostate Cancer DREAM Challenge, Kondofersky *et al.* ^42^ showed that employing simple recalibration models significantly improved prediction performance for subchallenge 1b. Moreover, researchers developed models specifically designed to amalgamate diverse patient cohorts by utilizing ideas from machine learning ^43– 45^.

## Conclusions

We proposed heterogeneous survival ensembles that are able to aggregate predictions from a wide variety of survival models. We evaluated our method using data from an independent fourth trial from the Prostate Cancer DREAM Challenge. Our proposed ensemble approach could predict the exact time of death more accurately than any competing model in sub challenge 1b and was significantly outperformed by 5 out of 50 competing solutions in sub challenge 1a. We believe this result is encouraging and warrants further research in using heterogeneous ensembles for survival analysis. The source code is available online https://www.synapse.org/#!Synapse:syn3647478.

## Supplementary material

Data pre-processing, missing imputations, and hyper-parameter configurations.
