# Supplementary material for: Heterogeneous ensembles for predicting survival of metastatic, castrate-resistant prostate cancer patients
Source: F1000Res. 2017 Jul 6;5:2676. Originally published 2016 Nov 16. [Version 3] doi: 10.12688/f1000research.8231.3 (PMC5500862; doi:10.12688/f1000research.8231.3)
Supplement: Supplementary file 1 [file f1000research-5-13099-s0000.tgz › b85d6b7c-240a-4115-ab62-7c705ae513f8.pdf]

Supplement: Heterogeneous Ensembles for Predicting Survival of Metastatic, Castrate-Resistant Prostate Cancer Patients

Sebastian Pölsterl[1], Pankaj Gupta[1], Lichao Wang[1], Sailesh Conjeti[1], Amin Katouzian[1], and Nassir Navab[1,2]

[1]Computer Aided Medical Procedures, Technische Universität München, Boltzmanstraße 3, 85748 Garching bei München, Germany
[2]Johns Hopkins University, 3400 North Charles Street, Baltimore, Maryland, 21218, USA

Contents

1 Data Pre-Processing 1
1.1 Subject-level Information (CoreTable) . . . 1
1.2 Prior Medications (PriorMed) . . . 1
1.3 Laboratory Tests (LabValue) . . . 2
1.4 Comorbidities (MedHistory) . . . 2
1.5 Lesion Measurements (LesionMeasure) . . . 2
1.6 Vital Signs (VitalSign) . . . 2
1.7 Post-Processing of Features . . . 2

2 Missing Value Imputation 2

3 Hyper-Parameter Configurations 3

1 Data Pre-Processing

Participants of the Prostate Cancer DREAM Challenge [1] were provided access to patients' health records from three separate phase III clinical trials [2, 3, 4] for training, and data from an independent, clinical trial of 470 men [5] for testing (values of dependent variables were held back and not revealed to participants). Data was organized into six tables comprising a wide range of clinical information: prior medications, comorbidities, target and non-target lesion measurements, laboratory tests, and vital signs. Five of six tables contained information at the event level and one table contained information that has been aggregated already. For instance, one table (`MedHistory`) listed all medical conditions one patient was subject to, while the total number and the type of conditions differed from patient to patient. Consequently, data from these tables had to be aggregated on a per-patient level to form a feature vector describing a single patient. In addition, inconsistencies in the terminology among all studies had to be discovered and resolved.

1.1 Subject-level Information (CoreTable)

Data in the CoreTable was already on a per-patient level, therefore no aggregation was necessary, instead we only formed new features:

1. Age, weight and height as categorical variables to be consistent with test data.
2. Visceral Metastases: Presence of visceral metastases was defined as presence of metastases in at least one of lungs, liver, adrenal gland, and pancreas.
3. Treatment: Indicator whether a patient was in the placebo group or treated with docetaxel or prednisone.

1.2 Prior Medications (PriorMed)

As the basis for features describing the use of medications prior to treatment, we used the chemical class of the drug (`cmatc4`) and the duration of medication (`CMDURN`, `CMSTDT_PC`, and `CMENDT_PC`). After resolving several inconsistencies among names of chemical classes across studies, we extracted features corresponding to the 60 most commonly used medications in the training data. A similar approach was used to derive features corresponding to the ten most common routes of administration (`CMROUTE`). Moreover, we added three more general features:

1. Whether a patient was undergoing hormone therapy before and for how long (`CMSCAT == "HORMONOTHERAPY"`).

2. Whether a patient was treated for an adverse event (CM_AE).
3. The total number of chemical classes a patient was subject to before treatment.

1.3 Laboratory Tests (LabValue)

Features regarding laboratory tests were extracted in a similar manner to prior medications above: we first resolved inconsistencies, and then obtained features corresponding to the 100 most performed tests (LBTESTCD) in the training data. For each lab test, we obtained its measurement (such as concentration), whether the measurement was within/below/above the reference range (LBSTNRLO and LBSTNRHI, if available), and when the test was performed first (LBDT_PC, relative to reference day).

1.4 Comorbidities (MedHistory)

Features derived from this table corresponded to the 20 most commonly encountered comorbidities in the training data (MHDECOD). Each comorbidity was associated with one binary feature denoting its presence or absence, and its start date (MHSTDT_P, relative to reference day). A feature with the total number of comorbidities per patient was added as well.

1.5 Lesion Measurements (LesionMeasure)

We extracted features from this table corresponding to the 20 most common locations of lesions (LSLOC2). Each location was described by a categorical feature denoting no lesion, 1 lesion, and 2 or more lesions, as well as the earliest day of assessment (LSDT_PC). Finally, we added the minimum and maximum lesion sizes, and the number of target and non-target lesions.

1.6 Vital Signs (VitalSign)

First, we ensured that height, weight, and temperature were measured on the same scale and then extracted features for all available vital signs from this table. In addition, we added the body mass index (BMI) as an additional feature.

1.7 Post-Processing of Features

To account for skewed distributions of features, especially with respect to laboratory measurements, we systematically checked the skewness of the empirical distribution and applied a log transformation if the skewness exceeded 1.4. Features corresponding to dates were transformed into four intervals by quantile-based discretization. Finally, we applied a Anscombe transformation [6] to features corresponding to counts.

The last step consisted of discarding useless features, i.e., those with one or less unique value, variance smaller than 0.001 (if continuous), or having all but one category that occurs more than ten times in the training data (if categorical).

2 Missing Value Imputation

As noted above, the Prostate Cancer DREAM Challenge was composed of data from four clinical studies. Therefore, which feature would be available for a patient depended on the study he or she participated in. We initially divided the data based on the common features available in the data of different studies. We considered a feature as unavailable if it was missing for more than 30% of patients in the respective study. In addition, we considered the union of two studies and all studies together, resulting in a total of 7 datasets as listed in table 1.

To impute missing values, we also considered features that were only available for a single study and, in particular, not for the challenge test data ("Features (Imputation)" in table 1). In contrast, when building models for the respective subchallenges, training data could only include features that were available in the challenge test data ("Features (Testing)" in table 1). We performed imputation based on random survival forests [7] to account for the remaining missing values. We started imputing the study-specific datasets (ASCENT-2, MAINSAIL, VENICE), because they had the most amount of features available, followed by imputing datasets consisting of multiple studies. The resulting data had no missing values and was used for all subsequent steps in our analyses. Continuous features were standardized to zero mean and unit variance.

For imputation of the challenge's test data, we applied the same scheme, except that we discarded features that were not available for the test data from the training data. With the remaining features, we trained a random survival forest [7] that subsequently was used to impute the test data (except its dependent variables).

ASCENT-2	MAINSAIL	VENICE	Samples	Features (Imputation)	Features (Testing)	Complete Cases
•	•	•	1,600	227	217	93.9%
	•	•	1,124	360	345	77.0%
•		•	1,074	230	220	92.1%
•	•		1,002	231	221	92.7%
		•	598	382	350	64.0%
	•		526	415	383	57.0%
•			476	242	223	78.8%

Table 1: Different sets of features that were constructed by considering the intersection between trials in the Prostate Cancer DREAM challenge. Features used during imputation can be absent in the test data (ENTHUSE-33 trial), whereas features for testing must be present in training and test data. Complete cases refers to the relative amount of samples free of missing values before imputation.

3 Hyper-Parameter Configurations

The list below provides a detailed list of hyper-parameter configurations used to build a heterogeneous ensemble of survival and regression models in the Prostate Cancer DREAM challenge.

- Cox proportional hazards model [8] with ridge penalty (13 configurations):
  - Penalty λ: $2^{-12}, 2^{-10}, \ldots, 2^{12}$
- Survival support vector machine [9] (13 configurations):
  - Penalty γ: $2^{-12}, 2^{-10}, \ldots, 2^{12}$
- Random survival forest [7] (24 configurations):
  - Number of of trees: 1,000
  - Minimum number of samples in a terminal node: 3, 5, 10, 25, 50, 100
  - Split criterion: log-rank splitting
  - Number of candidate splits to evaluate per feature: 2, 5, 10, ∞
- Gradient boosting with regression tree as base learner [10, 11] (1,728 configurations):
  - Number of iterations: 100, 500, 1000, 1500
  - Subsampling percentage: 100%, 75%, 50%
  - Learning rate: 0.06, 0.125, 0.25
  - Maximum number of leaf nodes: 5, 10, 20
  - Minimum number of samples per split: 2, 5, 10, 20
  - Maximum number of features to evaluate per split: all, $\sqrt{\#\text{ features}}$, 50%, 75%
- Gradient boosting with componentwise least squares as base learner [12, 10, 11] (36 configurations):
  - Number of iterations: 100, 500, 1000, 1500
  - Subsampling percentage: 100%, 75%, 50%
  - Learning rate: 0.06, 0.125, 0.25

References

[1] James Costello, Justin Guinney, Liz Zhou, Christopher Bare, Tao Wang, Brian Bot, Mike Kellen, Stephen Jones, Kalb Abdallah, Yang Xie, Elise Blaese, Joseph Morrell, Thea Norman, Stephen Friend, and Stolovitzkym Gustavo. Prostate Cancer DREAM Challenge.

[2] Howard I. Scher, Xiaoyu Jia, Kim Chi, Ronald de Wit, William R. Berry, Peter Albers, Brian Henick, David Waterhouse, Dean J. Ruether, Peter J. Rosen, Anthony A. Meluch, Luke T. Nordquist, Peter M. Venner, Axel Heidenreich, Luis Chu, and Glenn Heller. Randomized, open-label phase III trial of docetaxel plus high-dose calcitriol versus docetaxel plus prednisone for patients with castration-resistant prostate cancer. *Journal of Clinical Oncology: Official Journal of the American Society of Clinical Oncology*, 29(16):2191–2198, 2011.

[3] Daniel P Petrylak, Nicholas J Vogelzang, Nikolay Budnik, Pawel Jan Wiechno, Cora N Sternberg, Kevin Doner, Joaquim Bellmunt, John M Burke, Maria Ochoa de Olza, Ananya Choudhury, Juergen E Gschwend, Evgeny Kopyltsov, Aude Flechon, Nicolas Van As, Nadine Houede, Debora Barton, Abderrahim Fandi, Ulf Jungnelius, Shaoyi Li, Ronald de Wit, and Karim Fizazi. Docetaxel and prednisone with or without lenalidomide in chemotherapy-naive patients with metastatic castration-resistant prostate cancer (MAINSAIL): a randomised, double-blind, placebo-controlled phase 3 trial. *Lancet Oncology*, 16(4):417–425, 2015.

[4] Ian F Tannock, Karim Fizazi, Sergey Ivanov, Camilla Thellenberg Karlsson, Aude Fléchon, Iwona Skoneczna, Francisco Orlandi, Gwenaelle Gravis, Vsevolod Matveev, Sevil Bavbek, Thierry Gil, Luciano Viana, Osvaldo Arén, Oleg Karyakin, Tony Elliott, Alison Birtle, Emmanuelle Magherini, Laurence Hatteville, Daniel Petrylak, Bertrand Tombal, and Mark Rosenthal. Aflibercept versus placebo in combination with docetaxel and prednisone for treatment of men with metastatic castration-resistant prostate cancer (VENICE): a phase 3, double-blind randomised trial. *Lancet Oncology*, 14(8):760–768, 2013.

[5] Karim Fizazi, Celestia S Higano, Joel B Nelson, Martin Gleave, Kurt Miller, Thomas Morris, Faith E Nathan, Stuart McIntosh, Kristine Pemberton, and Judd W Moul. Phase III, randomized, placebo-controlled study of docetaxel in combination with zibotentan in patients with metastatic castration-resistant prostate cancer. *Journal of Clinical Oncology: Official Journal of the American Society of Clinical Oncology*, 31(14):1740–1747, 2013.

[6] F. J. Anscombe. The transformation of Poisson, binomial and negative-binomial data. *Biometrika*, 35(3-4):246–254, 1948.

[7] Hemant Ishwaran, Udaya B. Kogalur, Eugene H. Blackstone, and Michael S. Lauer. Random survival forests. *The Annals of Applied Statistics*, 2(3):841–860, 2008.

[8] D R Cox. Regression models and life tables. *Journal of the Royal Statistical Society: Series B*, 34:187–220, 1972.

[9] Sebastian Pölsterl, Nassir Navab, and Amin Katouzian. Fast training of support vector machines for survival analysis. In *Machine Learning and Knowledge Discovery in Databases*, volume 9285 of *Lecture Notes in Computer Science*, pages 243–259, 2015.

[10] Torsten Hothorn, Peter Bühlmann, Sandrine Dudoit, Annette Molinaro, and Mark J van der Laan. Survival ensembles. *Biostatistics*, 7(3):355–373, 2006.

[11] G. Ridgeway. The state of boosting. *Computing Science and Statistics*, 31:172–181, 1999.

[12] Peter Bühlmann and Bin Yu. Boosting with the L_2 loss. *Journal of the American Statistical Association*, 98(462):324–339, 2003.
