# Supplementary material for: Heterogeneous ensembles for predicting survival of metastatic, castrate-resistant prostate cancer patients
Source: F1000Res. 2017 Jul 6;5:2676. Originally published 2016 Nov 16. [Version 3] doi: 10.12688/f1000research.8231.3 (PMC5500862; doi:10.12688/f1000research.8231.3)
Supplement: Peer review history

# Peer review: Referee response for version 3

**DOI:** 10.5256/f1000research.13099.r24079

**Published:** 2017-07-25

**Authors:** Jinfeng Xiao

**Affiliations:** 1 Department of Computer Science, University of Illinois at Urbana–Champaign, Urbana, IL, USA

The authors have made a great revision and addressed all my concerns in my previous comments. The revised version is scientifically solid, and provides readers with a lot of insights and inspirations about the topic. Good job!

I have read this submission. I believe that I have an appropriate level of expertise to confirm that it is of an acceptable scientific standard.

# Peer review: Referee response for version 2

**DOI:** 10.5256/f1000research.13006.r23825

**Published:** 2017-06-28

**Authors:** Donna P. Ankerst

**Affiliations:** 1 Department of Mathematics, Technical University of Munich, Garching, Germany

The authors are to be congratulated for revising the article and thereby improving its important contribution to the science of prediction modeling. No further comments.

I have read this submission. I believe that I have an appropriate level of expertise to confirm that it is of an acceptable scientific standard.

# Peer review: Referee response for version 1

**DOI:** 10.5256/f1000research.8853.r19237

**Published:** 2017-03-14

**Authors:** Amber L Simpson

**Affiliations:** 1 Department of Surgery, Memorial Sloan Kettering Cancer Center, New York, NY, USA

The authors describe an ensemble approach for predicting survival in prostate cancer patients as part of the 2015 Prostate Cancer DREAM (Dialogue for Reverse Engineering Assessments and Methods) Challenge. Patients included in the study had metastatic, castrate-resistant prostate cancer, an advanced cancer with poor prognosis. The authors are commended for undertaking a difficult problem and providing an elegant solution incorporating Cox, gradient boosting, random survival forest, and SVM for time-dependent analysis.

 Addressing these points would improve the manuscript:

 1) The luxury of a challenge is that authors are positioned to use knowledge gained from the challenge to improve their prediction model. The intent of sharing these datasets is to develop the best biomarker that can be used to change patient selection for therapy. Can the authors comment on what they would do differently now that they have considered methods proposed by other groups in the challenge? How can others use the lessons learned in this challenge to make the best biomarker possible?

 2) The authors should also comment on the generalizability of their methods to other problems.

 3) The paper is a good technical companion paper to the overview paper that was recently released, which should be cited ^1^.

 4) For those unfamiliar with the challenge, it is important to note that the challenge organizers confirmed performance on the validation data as noted by a reviewer above. This information should be incorporated into the manuscript, as it is not readily apparent.

 5) How does the model perform relative to published clinical nomograms? For example, the Armstrong nomogram achieved a concordance index of 0.69. Can the authors comment on the improvement over existing methods? One could argue that the slight improvement is not worth the overhead of employing ensemble methods ^2^^-^^4^

 6) How does predicting survival change the management of these patients? For example, would bad actors be selected for a different treatment or spared from treatment? If so, it may be appropriate to calculate positive and negative predictive value for specific time points. Maximizing positive and negative predictive value may also make sense. The proposed method could aid in chemoprevention, as an example.

 7) Is it possible to make the model publicly available as a nomogram (see nomograms.org)? Clinicians will not have the ability to download and install the code, but they may be interested in the results for individual patients.

 8) How does the ensemble method compensate for highly correlated variables?

 9) How was feature selection performed?

 10) Listing the features would be helpful for clinicians looking to refine/improve existing nomograms.

I have read this submission. I believe that I have an appropriate level of expertise to confirm that it is of an acceptable scientific standard, however I have significant reservations, as outlined above.

## References

[1] GuinneyJWangTLaajalaTWinnerKBareJNetoEKhanSPeddintiGAirolaAPahikkalaTMirttiTYuTBotBShenLAbdallahKNormanTFriendSStolovitzkyGSouleHSweeneyCRyanCScherHSartorOXieYAittokallioTZhouFCostelloJ: Prediction of overall survival for patients with metastatic castration-resistant prostate cancer: development of a prognostic model through a crowdsourced challenge with open clinical trial data. *The Lancet Oncology*.2017;18(1) : 10.1016/S1470-2045(16)30560-5 132-142 10.1016/S1470-2045(16)30560-5 PMC521718027864015

[2] ArmstrongAJGarrett-MayerESYangYCde WitRTannockIFEisenbergerM: A contemporary prognostic nomogram for men with hormone-refractory metastatic prostate cancer: a TAX327 study analysis. *Clin Cancer Res*.2007;13(21) : 10.1158/1078-0432.CCR-07-1036 6396-403 10.1158/1078-0432.CCR-07-1036 17975152

[3] ArmstrongAJGarrett-MayerEde WitRTannockIEisenbergerM: Prediction of survival following first-line chemotherapy in men with castration-resistant metastatic prostate cancer. *Clin Cancer Res*.2010;16(1) : 10.1158/1078-0432.CCR-09-2514 203-11 10.1158/1078-0432.CCR-09-2514 20008841

[4] HalabiSLinCYKellyWKFizaziKSMoulJWKaplanEBMorrisMJSmallEJ: Updated prognostic model for predicting overall survival in first-line chemotherapy for patients with metastatic castration-resistant prostate cancer. *J Clin Oncol*.2014;32(7) : 10.1200/JCO.2013.52.3696 671-7 10.1200/JCO.2013.52.3696 24449231PMC3927736

# Response

**Published:** 2017-06-22

**Authors:** Sebastian Pölsterl

**Affiliations:** Technische Universität München, Germany

The authors describe an ensemble approach for predicting survival in prostate cancer patients as part of the 2015 Prostate Cancer DREAM (Dialogue for Reverse Engineering Assessments and Methods) Challenge. Patients included in the study had metastatic, castrate-resistant prostate cancer, an advanced cancer with poor prognosis. The authors are commended for undertaking a difficult problem and providing an elegant solution incorporating Cox, gradient boosting, random survival forest, and SVM for time-dependent analysis.

 Addressing these points would improve the manuscript:

1. The luxury of a challenge is that authors are positioned to use knowledge gained from the challenge to improve their prediction model. The intent of sharing these datasets is to develop the best biomarker that can be used to change patient selection for therapy. Can the authors comment on what they would do differently now that they have considered methods proposed by other groups in the challenge? How can others use the lessons learned in this challenge to make the best biomarker possible?
  1. ***Response*** *: Several teams, including the winning team (FIMM-UTU), implemented methods to carefully select patients from the three studies constituting the training data such that they are not too different from the target study, which was used for the final evaluation. We believe that a considerable improvement can be gained by discarding outliers from the training data.*
2. The authors should also comment on the generalizability of their methods to other problems.
  1. ***Response*** *: Our proposed solution relies on heterogeneous ensembles, which are comprised of survival models to predict the risk of death. Hence, our approach is directly applicable to any data with right censored survival times. For other problems, such as classification or regression, the ensemble selection and ensemble pruning need to be adapted by choosing an appropriate performance measure (see line 11 of algorithm 1 and line 14 of algorithm 2). In fact, the original authors of heterogeneous ensembles investigated ten performance metrics for classification and Rooney et al. proposed to using the mean squared error for regression problems. Therefore, heterogeneous ensembles are applicable to a wide range of learning problems.*
3. The paper is a good technical companion paper to the overview paper that was recently released, which should be cited ^1^.
  1. ***Response*** *: We updated reference 16 to refer to the paper in The Lancet Oncology.*
4. For those unfamiliar with the challenge, it is important to note that the challenge organizers confirmed performance on the validation data as noted by a reviewer above. This information should be incorporated into the manuscript, as it is not readily apparent.
  1. ***Response*** *: We updated the last paragraph of the “Validation scheme” section and the first paragraph of the “Challenge hold-out data” to emphasize that validation was carried out by the challenge organizers.*
5. How does the model perform relative to published clinical nomograms? For example, the Armstrong nomogram achieved a concordance index of 0.69. Can the authors comment on the improvement over existing methods? One could argue that the slight improvement is not worth the overhead of employing ensemble methods ^2^ ^-^ ^4^
  1. ***Response*** *: In subchallenge 1a, submissions of all participating teams were compared to the model by Halabi et al., which was considered the state-of-the-art risk prediction model prior to the challenge. Only submissions with a statistically better performance than the model by Halabi et al. were considered for the final evaluation (see section Validation scheme in our manuscript for further details). Our proposed model achieved an iAUC score of 0.7646 on the challenge’s hold data, whereas the model by Halabi et al. achieved a score of 0.7432, which is significantly worse: the Bayes factor of the proposed model vs. Halabi et al. model, is 12.2, which indicates strong evidence.*
6. How does predicting survival change the management of these patients? For example, would bad actors be selected for a different treatment or spared from treatment? If so, it may be appropriate to calculate positive and negative predictive value for specific time points. Maximizing positive and negative predictive value may also make sense. The proposed method could aid in chemoprevention, as an example.
  1. ***Response*** *: We agree that ultimately the focus should be on improving patient treatment, but at the same time computational methods can only hint at potentially interesting biomarkers or patient subgroups, whether this information is useful in the clinic requires additional research, e.g., to rule-out harmful side-effects. Data in the Prostate Cancer DREAM Challenge are collated based on comparator arm data sets of Phase III prostate cancer clinical trials, where all patients received docetaxel and prednisone in the comparator arm. Therefore, we could not determine whether differences in survival can be attributed to different treatment types. If outcome information from multiple treatments were available, it would indeed be very interesting to infer the optimal treatment by maximizing positive and negative predictive value over time instead of specificity and sensitivity as the iAUC metric used in the challenge does.*
7. Is it possible to make the model publicly available as a nomogram (see nomograms.org)? Clinicians will not have the ability to download and install the code, but they may be interested in the results for individual patients.
  1. ***Response*** *: Unfortunately, it is often difficult to understand how an ensemble method relates the input to variables to each other in order to form a prediction, which is especially true for heterogeneous ensembles, because of their non-linear nature. A nomogram describes a non-linear model only inadequately, because it gives each variable only a single weight and usually lacks high-order interactions. However, there are several alternative ways to obtain insight. For instance, Breiman (Machine Learning, 45:1, 2001.* *http://dx.doi.org/10.1023/A:1010933404324* *) suggested a variable importance measure for random forests that could be adapted. The j-th feature is randomly permuted for all out-of-bag samples and run down the corresponding tree. The output is the relative increase in prediction error as compared to if the j-th feature is intact. Feature with a larger increase in prediction error, are considered more important to the ensemble. If one wants to infer which interactions among features the ensemble considers, more sophisticate methods are available (e.g. Henelius et al., SLDS 2015.* *http://dx.doi.org/10.1007/978-3-319-17091-6_5)* *.*
8. How does the ensemble method compensate for highly correlated variables?
  1. ***Response*** *: Whether the ensemble compensates for highly correlated variables depends on choice of base learners. Here, all base learners account for multicolinearities. The penalized Cox model and survival support vector machine use a ridge (L2) penalty, gradient boosting with regression trees and random survival forests recursively split the data based on a single feature, and gradient boosting with componentwise least squares selects only one feature in each iteration such that the error is maximally reduced. Hence, all models can be trained despite highly correlated variables in the data.*
9. How was feature selection performed?
  1. ***Response*** *: We did not perform feature selection prior to constructing the heterogeneous ensemble, however, the ensemble comprised base learners that implicitly perform feature selection when trained on high-dimensional data, namely random survival forest and gradient boosting models. The remaining models (penalized Cox model and survival support vector machine) do not perform feature selection and only account for multicolinearities.*
10. Listing the features would be helpful for clinicians looking to refine/improve existing nomograms.
  1. ***Response*** *: We trained models on different subsets of the data, ranging from 383 features for data from the MAINSAIL trial to 217 features when combining data of all three trials (see table 1). More details on the extracted features are available from the supplementary material and at* *https://www.synapse.org/#!Synapse:syn4650470* *.*

# Peer review: Referee response for version 1

**DOI:** 10.5256/f1000research.8853.r20214

**Published:** 2017-03-06

**Authors:** Jinfeng Xiao

**Affiliations:** 1 Department of Computer Science, University of Illinois at Urbana–Champaign, Urbana, IL, USA

This paper is written by CAMP, a winning team of the 2015 Prostate Cancer DREAM Challenge (“the PCDC”, or “the challenge”), to introduce their winning method. The authors built heterogeneous ensembles with the training data (Trials ASCENT-2, MAINSAIL, VENICE) and the unlabeled part of the validation data (Trial ENTHUSE-33). The high performance of their method, especially in predicting patients’ days to death, was confirmed by the challenge organizers on the validation data. This manuscript contains sufficient details about the actual method they used in the PCDC. Achilles heels for this paper include: 1) To show the necessity of using ensembles; 2) To establish the generalizability of the proposed ensemble models to new data sets. See the major issues below for more detailed comments.

**Major issues**:

1. The power of averaging over the base learners was taken for granted in the paper without experimental evidence. Training an ensemble costs much more effort than training a single model, and therefore it has to be shown that such effort is worth it. Direct comparison in performance between the ensemble and base learners is needed to make this point clear.
2. The training of the ensembles, in particular the ensemble pruning step, used information from the validation set. Although only the features, but not the outcomes, of the validation data were seen by the model, this practice is still not encouraged. A generalizable model should not use the validation data in any way during training. Therefore, whether the proposed method is generalizable to new data sets is in doubt. I would suggest the authors to prune the ensemble on the training set and check the performance on the validation set.
3. There is no instruction in the code documentation about how to apply the code to new data sets. Adding such information can greatly increase the chance that the code will be used by other researchers.
4. Can the authors mine some knowledge from the trained model? For example, what are the most important features? Where are the baseline (i.e. Halabi’s model ^1^) features in the ranked list? Such analysis of the model can be helpful to biomedical researchers and doctors.

 

**Minor issues**:

1. In Algorithms 1 & 2, how did the authors choose the minimum desired performance c _min_ and the desired set of ensemble S?
2. Page 6, paragraph 2, line 3: “Median” should be changed to “standard deviation” or some other measures of variance, because in a within-trial validation the “median” is not directly related to “the difference between observed time points in the training and test data” (lines 5-6).
3. Page 8, paragraph 2, the last 8 lines: This example is not very convincing. A model considering all features trained on the first dataset will assign a very small (if not zero) weight to feature 3, which will compensate little for the fact that feature 3 is important in the second dataset.
4. Page 8, paragraph 5: What numerical difficulties did the authors encounter so that they could not include the Cox regression in the ensembles? Is there anything special about Cox model that makes it harder to train than other base learners?
5. It is not explicitly stated in the paper that the authors are from Team CAMP.

 

**Grammar**:

 Page 4, last paragraph: “within-in trial validation” should be “within-trial validation”; “between trials validation” should be “between-trial validation”.

I have read this submission. I believe that I have an appropriate level of expertise to confirm that it is of an acceptable scientific standard, however I have significant reservations, as outlined above.

## References

[1] HalabiSLinCYKellyWKFizaziKSMoulJWKaplanEBMorrisMJSmallEJ: Updated prognostic model for predicting overall survival in first-line chemotherapy for patients with metastatic castration-resistant prostate cancer. *J Clin Oncol*.2014;32(7) : 10.1200/JCO.2013.52.3696 671-7 10.1200/JCO.2013.52.3696 24449231PMC3927736

# Response

**Published:** 2017-06-22

**Authors:** Sebastian Pölsterl

**Affiliations:** Technische Universität München, Germany

This paper is written by CAMP, a winning team of the 2015 Prostate Cancer DREAM Challenge (“the PCDC”, or “the challenge”), to introduce their winning method. The authors built heterogeneous ensembles with the training data (Trials ASCENT-2, MAINSAIL, VENICE) and the unlabeled part of the validation data (Trial ENTHUSE-33). The high performance of their method, especially in predicting patients’ days to death, was confirmed by the challenge organizers on the validation data. This manuscript contains sufficient details about the actual method they used in the PCDC. Achilles heels for this paper include: 1) To show the necessity of using ensembles; 2) To establish the generalizability of the proposed ensemble models to new data sets. See the major issues below for more detailed comments.

**Major issues**:

1. The power of averaging over the base learners was taken for granted in the paper without experimental evidence. Training an ensemble costs much more effort than training a single model, and therefore it has to be shown that such effort is worth it. Direct comparison in performance between the ensemble and base learners is needed to make this point clear.
  1. ***Response*** *:* *We included heterogeneous ensembles in the between trials validation (see figures 3 and 5) and in our discussion of the results.*
2. The training of the ensembles, in particular the ensemble pruning step, used information from the validation set. Although only the features, but not the outcomes, of the validation data were seen by the model, this practice is still not encouraged. A generalizable model should not use the validation data in any way during training. Therefore, whether the proposed method is generalizable to new data sets is in doubt. I would suggest the authors to prune the ensemble on the training set and check the performance on the validation set.
  1. ***Response*** *: For survival data, the pruning step is delayed until prediction is performed, because predictions are risk scores on an arbitrary scale for which a per-sample error measure is not readily available. This is in contrast to ensemble pruning for regression problems, where a per-sample error can be easily computed and models having highly correlated errors are pruned. As referee 2 suggests, the pruning step could be performed via cross-validation on the training data. However, our pruning step does not take survival times or censoring status into account, therefore we prefer to delay the pruning step as long as possible as to avoid overfitting on the training data. If the additional costs associated with storing the ensemble before pruning are prohibitive, we recommend that pruning should be performed via cross-validation on the training data.*
3. There is no instruction in the code documentation about how to apply the code to new data sets. Adding such information can greatly increase the chance that the code will be used by other researchers.
  1. ***Response:*** *Our source code is accompanied by a README file explaining all steps necessary to reproduce our results. The source code can be downloaded from Synapse (* *http://dx.doi.org/10.7303/syn3647478* *) as well as GitHub (* *https://github.com/tum-camp/dream-prostate-cancer-challenge* *).*
4. Can the authors mine some knowledge from the trained model? For example, what are the most important features? Where are the baseline (i.e. Halabi’s model ^1^) features in the ranked list? Such analysis of the model can be helpful to biomedical researchers and doctors.
  1. ***Response*** *: Please see our response to question 8 of referee 1.*

**Minor issues**:

1. In Algorithms 1 & 2, how did the authors choose the minimum desired performance c _min_ and the desired set of ensemble S?
  1. ***Response*** *: We chose c*_*min*_ *= 0.66 based on results of the with-in trial validation (figure 2): approximately 30% of the experiments performed worse. The final ensemble consists of all base learners in the top 5% according the combined accuracy and diversity score (see table 2 and algorithm 2). Both c*_*min*_ *and S remained fixed throughout our experiments and were not optimised.*
2. Page 6, paragraph 2, line 3: “Median” should be changed to “standard deviation” or some other measures of variance, because in a within-trial validation the “median” is not directly related to “the difference between observed time points in the training and test data” (lines 5-6).
  1. ***Response*** *: Thank you for the suggestion, we replaced median by standard deviation in the manuscript.*
3. Page 8, paragraph 2, the last 8 lines: This example is not very convincing. A model considering all features trained on the first dataset will assign a very small (if not zero) weight to feature 3, which will compensate little for the fact that feature 3 is important in the second dataset.
  1. ***Response*** *:* *We agree that the example was inadequate to explain this observation. We replaced it by referencing the work by Meinshausen and Bühlmann, who showed that models with embedded feature selection suffer from false positive selections in high dimensions.*
4. Page 8, paragraph 5: What numerical difficulties did the authors encounter so that they could not include the Cox regression in the ensembles? Is there anything special about Cox model that makes it harder to train than other base learners?
  1. ***Response*** *: Please see our response to question 7 of referee 1.*
5. It is not explicitly stated in the paper that the authors are from Team CAMP.
  1. ***Response*** *: We mentioned that we participated under the name “Team CAMP” at the end of the introduction and in the section “Challenge hold-out data”.*

**Grammar**:

 Page 4, last paragraph: “within-in trial validation” should be “within-trial validation”; “between trials validation” should be “between-trial validation”.

***Response*** *: Thank you, we corrected these errors in the manuscript.*

# Peer review: Referee response for version 1

**DOI:** 10.5256/f1000research.8853.r18609

**Published:** 2016-12-19

**Authors:** Donna P. Ankerst

**Affiliations:** 1 Department of Mathematics, Technical University of Munich, Garching, Germany

The authors are to be congratulated for landing among the circle of winners of the Prostate Cancer DREAM Challenge and for clearly describing their innovative methods in this paper. An informative discussion critically appraises their approach, providing suggestions for advancing the field of clinical risk prediction. Instead of relying on one survival model, their approach hinges on heterogeneous ensembles that invoke a variety of model types, including gradient boosting (least squares versus trees), random survival forests, and survival support vector machines (linear versus clinical kernels), thereby hedging against sub-optimality of any single model for any single test set. I have only minor comments.

1. It is argued throughout that heterogeneous ensembles have been shown to be optimal compared to single models for this challenge, but I did not see a head-to-head comparison illustrating this. For example, could one not add ensemble methods as an extra column to the within- and between-trial validations in Figures 2 and 3, respectively?
2. I greatly appreciated Figure 4 that showed which of the multiple comparisons in Figure 3 (the between-trial validation) were actually critically different, as many of the iAUCs only differed out to the second decimal (which is by the way a clinically meaningless difference). It would be nice to also have such a comparison for Figure 2 (the within-trial validation) that could definitely show whether or not the Cox model was statistically indistinguishable from random forests, and to temper the Results section concerning the comparison of the methods. One method only beats another if the confidence intervals of the respective AUCs do not overlap. Given their similar performance, the comparison among the different individual survival models might not be as relevant as whether or not the ensemble outperformed any one of them.
3. As nicely pointed out in the Discussion, it is a surprise and a great pity that the concordance statistic c was used for the training of the models instead of the iAUC, the criterion used for evaluation for the challenge. While easy to compute, the concordance statistic suffers greatly from censored observations, they essentially are discarded in the evaluation. This means that only a minority of the data in the ASCENT and MAINSAIL trials were used (71% and 82.5% of the data censored). The iAUC, however, also suffers from censored data, but from what I understand, to a lesser extent. Is it possible to redo Figures 2 and 3 using the iAUC instead of the concordance statistic, to see if similar conclusions held?
4. In the discussion of the within-trial internal cross-validation of Figure 2 it is mentioned that some of the methods may have performed poorly because of a difference in follow-up between the random partitions of the trial into training and test sets. In medical studies, this is often controlled using stratified randomization, which ensures the proportion of observed events (deaths in this case) or follow-up remains equal across the sets. Would it be possible to implement to see if it improved the outcome for VENICE, in order to help explain the poor behavior there? It of course, does not help the between-trial validation, the subject of the next point.
5. The problem of recalibration to different trials is becoming more and more recognized in medicine; searching for “recalibration risk score” or “recalibration risk model” in PubMed reveals hundreds of suggestions and applications. The authors do a nice job of illustrating the particular difficulties with survival data – a look at Figure 1 shows that median follow-up in the held out ENTHUSE-33 trial was longer than two of the trials used for training. In our analysis for the challenge we showed that recalibration made a big difference for the root-mean-squared-error in Subchallenge 1b but not the iAUC in Subchallenge 1a, matching previous results we have obtained in proposals to dynamically update risk models ( https://www.ncbi.nlm.nih.gov/pmc/articles/PMC4532612/). Recalibration means any method to tweak an existing risk model to conform to a particular target population, but has the problem that it requires data from the intended target population, something not generally possible for clinical practice. I agree with the authors that this could have improved their models and would like to see more discussion of the literature from recalibration of survival models.
6. In the Discussion of the between-trials validation, the authors try to explain the surprising result that the simpler Cox model with its stringent proportional hazards and linear assumption performs as well as some of the other models that incorporate non-linearity. I think lack of statistical power, i.e., small sample size, may be another culprit here. The effective information size for survival data (defined as the size of the information matrix) is only proportional to the number of observed events and not the total sample size, this is an issue that clinical trial statisticians who design trials understand well, but unfortunately not the rest of the community. It was a point I tried to raise at the first Challenge webinar, foreseeing that there would be many ties among winners due to the high censoring. While for training it seemed like there were trials of size 476, 526 and 598 patients for the respective trials in Figure 1, with a total of 1600 patients, the effective information content was only 138, 92, and 432, respectively, for a  total of 662 patients. Simulation studies would reveal what sample size would be needed to detect nonlinearities of different magnitudes. My point is not to suggest doing these, but rather to modify the discussion that the high-performance of Cox’s simpler model may be due to the Occam’s Razor principle, that if there exists two explanations for data, the simpler is preferred.
7. In light of Point 6.), it is a pity that the well-performing Cox’s proportional hazards model was eventually dropped because of numerical problems. Our team used this model without much difficulty. Can the authors elaborate here or propose suggestions for overcoming the numerical difficulties? For example, could it be that the input data contained a lot of features with anomalies that should have been cleaned out?
8. I realize it was not the point of this paper, but it is a pity that there is no discussion of the specifics of the 90 features that ultimately made it into the prediction models. Were they the same as the ones found by Halabi *et al.*? 90 features are a lot and not generally implementable in online risk tools designed to help patients – would there be a way to summarize the features that are most important in order to help clinicians understand the important indicators?
9. Looking back at the Halabi paper, which has a simple Cox model with a handful of predictors that is immediately interpretable, the AUC obtained there on the test set (0.76) seems close to those obtained in this challenge. The AUC is a rank-based discrimination measure, that reflects the probability that for a randomly selected pair of patients, the patient that died later had a lower risk score and differences have to be interpreted relative to this meaning. I would like to hear the authors’ reflection as to whether the DREAM Challenge has proven the case for the large-scale methods used in the Challenge or against them. What future directions are needed to improve prediction? Some, like myself, would argue that new markers need to be discovered rather than bigger models.

I have read this submission. I believe that I have an appropriate level of expertise to confirm that it is of an acceptable scientific standard, however I have significant reservations, as outlined above.

# Response

**Published:** 2017-06-22

**Authors:** Sebastian Pölsterl

**Affiliations:** Technische Universität München, Germany

The authors are to be congratulated for landing among the circle of winners of the Prostate Cancer DREAM Challenge and for clearly describing their innovative methods in this paper. An informative discussion critically appraises their approach, providing suggestions for advancing the field of clinical risk prediction. Instead of relying on one survival model, their approach hinges on heterogeneous ensembles that invoke a variety of model types, including gradient boosting (least squares versus trees), random survival forests, and survival support vector machines (linear versus clinical kernels), thereby hedging against sub-optimality of any single model for any single test set. I have only minor comments.

1. It is argued throughout that heterogeneous ensembles have been shown to be optimal compared to single models for this challenge, but I did not see a head-to-head comparison illustrating this. For example, could one not add ensemble methods as an extra column to the within- and between-trial validations in Figures 2 and 3, respectively?
  1. ***Response*** *: We included heterogeneous ensembles in the between trials validation (see figures 3 and 5) and in our discussion of the results.*
2. I greatly appreciated Figure 4 that showed which of the multiple comparisons in Figure 3 (the between-trial validation) were actually critically different, as many of the iAUCs only differed out to the second decimal (which is by the way a clinically meaningless difference). It would be nice to also have such a comparison for Figure 2 (the within-trial validation) that could definitely show whether or not the Cox model was statistically indistinguishable from random forests, and to temper the Results section concerning the comparison of the methods. One method only beats another if the confidence intervals of the respective AUCs do not overlap. Given their similar performance, the comparison among the different individual survival models might not be as relevant as whether or not the ensemble outperformed any one of them.
  1. ***Response*** *: As suggested, we added a plot for the results presented in figure 2. It shows that the Cox model and random survival forest only significantly outperform linear SVM, whereas the performance of the other methods lies within the critical difference interval.*
3. As nicely pointed out in the Discussion, it is a surprise and a great pity that the concordance statistic c was used for the training of the models instead of the iAUC, the criterion used for evaluation for the challenge. While easy to compute, the concordance statistic suffers greatly from censored observations, they essentially are discarded in the evaluation. This means that only a minority of the data in the ASCENT and MAINSAIL trials were used (71% and 82.5% of the data censored). The iAUC, however, also suffers from censored data, but from what I understand, to a lesser extent. Is it possible to redo Figures 2 and 3 using the iAUC instead of the concordance statistic, to see if similar conclusions held?
  1. ***Response*** *: We did perform the same analyses as depicted in figures 2 and 3 using iAUC as evaluation criteria. When ranking methods according to average iAUC, one arrives at the same conclusion as when ranking according to average c-index. However, the average performance with respect to the test datasets are quite different. As we pointed out in the main text, this is due to the definition of the iAUC used in the Prostate Cancer Dream Challenge, which is the integral over time points every 6 months up to 30 months after the first day of treatment. This would cover most time points in ASCENT-2 and MAINSAIL, but would miss out many events occurring after 30 months for VENICE (cf. figure 1). Consequently, it appears that all methods perform considerably better when tested on the VENICE data. Usually, it is recommended to choose the limits of the interval to integrate over from the data, e.g. the 5% to 90% percentile of observed time points. However, the iAUC would be based on a different interval for each study, making inter-study comparisons difficult to interpret. Therefore, we believe that the c-index is easier to interpret when considering the inter-study comparison. In addition, we re-trained our heterogeneous ensemble using the iAUC metric in algorithm 2 and submitted its prediction after the challenge concluded. We obtained an iAUC of 0.7636 compared to 0.7644 when using c-index, and 0.7537 for the Halabi model.*
4. In the discussion of the within-trial internal cross-validation of Figure 2 it is mentioned that some of the methods may have performed poorly because of a difference in follow-up between the random partitions of the trial into training and test sets. In medical studies, this is often controlled using stratified randomization, which ensures the proportion of observed events (deaths in this case) or follow-up remains equal across the sets. Would it be possible to implement to see if it improved the outcome for VENICE, in order to help explain the poor behavior there? It of course, does not help the between-trial validation, the subject of the next point.
  1. ***Response*** *: We implemented the suggested modification to perform stratified cross-validation and repeated the experiment. The results are very similar to figure 2: the average performance of all methods is still worst when trained and tested on data from the VENICE study.*
5. The problem of recalibration to different trials is becoming more and more recognized in medicine; searching for “recalibration risk score” or “recalibration risk model” in PubMed reveals hundreds of suggestions and applications. The authors do a nice job of illustrating the particular difficulties with survival data – a look at Figure 1 shows that median follow-up in the held out ENTHUSE-33 trial was longer than two of the trials used for training. In our analysis for the challenge we showed that recalibration made a big difference for the root-mean-squared-error in Subchallenge 1b but not the iAUC in Subchallenge 1a, matching previous results we have obtained in proposals to dynamically update risk models ( https://www.ncbi.nlm.nih.gov/pmc/articles/PMC4532612/). Recalibration means any method to tweak an existing risk model to conform to a particular target population, but has the problem that it requires data from the intended target population, something not generally possible for clinical practice. I agree with the authors that this could have improved their models and would like to see more discussion of the literature from recalibration of survival models.
  1. ***Response*** *: We agree with the referee that calibration is an important aspect besides discrimination that should be considered for prognostic models. The focus of the referenced article is on calibration of binary classification models, which is very different from calibration with respect to survival models. In contrast to binary classification, predicted risk scores of a survival model are only the relative risk, but not absolute risks (Royston and Altman. BMC Medical Research Methodology, 13(1), 2013.* *http://doi.org/10.1186/1471-2288-13-33* *). Although, a measure of absolute risk can be derived for the Cox model by estimating the baseline hazard function, to the best of our knowledge, there is no standard approach to statistically assess calibration for arbitrary survival models. We could only visually assess calibration by constructing low, medium, and high risk groups corresponding to cut-offs at the 33% and 66% percentile of predicted risk scores. Using the same cut-offs on predicted risk scores from hold-out dataset, we constructed two Kaplan-Meier plots, each stratified by risk group. For a well calibrated model, we would expect the Kaplan-Meier curves derived from the training and hold-out data to agree with each other. The disadvantage of this approach is that we cannot precisely quantify the lack of calibration.*
6. In the Discussion of the between-trials validation, the authors try to explain the surprising result that the simpler Cox model with its stringent proportional hazards and linear assumption performs as well as some of the other models that incorporate non-linearity. I think lack of statistical power, i.e., small sample size, may be another culprit here. The effective information size for survival data (defined as the size of the information matrix) is only proportional to the number of observed events and not the total sample size, this is an issue that clinical trial statisticians who design trials understand well, but unfortunately not the rest of the community. It was a point I tried to raise at the first Challenge webinar, foreseeing that there would be many ties among winners due to the high censoring. While for training it seemed like there were trials of size 476, 526 and 598 patients for the respective trials in Figure 1, with a total of 1600 patients, the effective information content was only 138, 92, and 432, respectively, for a  total of 662 patients. Simulation studies would reveal what sample size would be needed to detect nonlinearities of different magnitudes. My point is not to suggest doing these, but rather to modify the discussion that the high-performance of Cox’s simpler model may be due to the Occam’s Razor principle, that if there exists two explanations for data, the simpler is preferred.
  1. ***Response*** *: Thank you for pointing out that effective sample size could be limiting the* *performance* *of more complicated models. We added a paragraph discussing this issue to the “Between trials validation” section.*
7. In light of Point 6.), it is a pity that the well-performing Cox’s proportional hazards model was eventually dropped because of numerical problems. Our team used this model without much difficulty. Can the authors elaborate here or propose suggestions for overcoming the numerical difficulties? For example, could it be that the input data contained a lot of features with anomalies that should have been cleaned out?
  1. ***Response*** *: We fit Cox’s proportional hazards model using a Newton-Rhapson algorithm with constant step size, i.e., without a line search. We observed that optimization sometimes diverged, which can be attributed to choosing a constant step size. If the chosen step size is too large, it can lead to oscillation around the minimum due to overshooting and the minimum is never reached. A crude solution would be to increase the tolerance that determines convergence or choose a different starting point. A better solution would be to determine the optimal step size in each iteration of Newton’s method via line search or employing a trust region method (see e.g. Boyd and Vandenberghe, Convex Optimization, Cambridge University Press, 2009). Unfortunately, we were unable to implement this modification before the challenge was closed.*
8. I realize it was not the point of this paper, but it is a pity that there is no discussion of the specifics of the 90 features that ultimately made it into the prediction models. Were they the same as the ones found by Halabi *et al.*? 90 features are a lot and not generally implementable in online risk tools designed to help patients – would there be a way to summarize the features that are most important in order to help clinicians understand the important indicators?
  1. ***Response*** *: Our final ensemble considered 217 features (see table 1), which included those found by Halabi et al., and was comprised of 90 different base learners (see table 3). We agree that the raw predictive performance of a model often provides insufficient information in medical research. Clearly, there is a trade-off between model complexity and how well a model can be interpreted. As we mentioned in the introduction of our manuscript, an ensemble approach is only beneficial if base learners perform better than random prediction and are diverse. The latter can be achieved by using base learners that are based on different loss functions (heterogeneous ensemble) or by using the same loss for all base learners and forcing each base learner to consider different subsets of features (homogeneous ensemble). We chose to utilize both types by selecting base learners from a large library of different models and identical, but differently parametrized, models. Consequently, we encourage base learners to weight features differently, which makes creating a universal ranking of features challenging. Although feature importances are not directly available from our final ensemble, there are several alternative ways to obtain insight. For instance, Breiman (Machine Learning, 45:1, 2001.* *http://dx.doi.org/10.1023/A:1010933404324* *) suggested a variable importance measure for random forests that could be adapted. The j-th feature is randomly permuted for all out-of-bag samples and run down the corresponding tree. The output is the relative increase in prediction error as compared to if the j-th feature is intact. Feature with a larger increase in prediction error, are considered more important to the ensemble. If one wants to infer which interactions among features the ensemble considers, more sophisticate methods are available (e.g. Henelius et al., SLDS 2015.* *http://dx.doi.org/10.1007/978-3-319-17091-6_5)* *.*
9. Looking back at the Halabi paper, which has a simple Cox model with a handful of predictors that is immediately interpretable, the AUC obtained there on the test set (0.76) seems close to those obtained in this challenge. The AUC is a rank-based discrimination measure, that reflects the probability that for a randomly selected pair of patients, the patient that died later had a lower risk score and differences have to be interpreted relative to this meaning. I would like to hear the authors’ reflection as to whether the DREAM Challenge has proven the case for the large-scale methods used in the Challenge or against them. What future directions are needed to improve prediction? Some, like myself, would argue that new markers need to be discovered rather than bigger models.
  1. ***Response*** *: The Prostate Cancer DREAM* *challenge provided* *high-quality data to a large group of researchers, which led to improved prediction performance compared to the model by Halabi et al. and highlighted interesting problems for future research. In particular, we believe that future research should focus on how to best utilize data from multiple clinical trials and how to adapt a model to new patient cohorts. As described in our manuscript, the Prostate Cancer DREAM* *challenge* *compiled data from four clinical trials, with each trial having its own characteristics, ranging from different follow-up periods to different clinical information collected. In light of these differences, just combining all the data and learning a model on top of it is likely to lead to a poor model, despite an increase in sample size. Multiple teams identified this problem and tried to address it. Most importantly, the winning team (FIMM-UTU) selected a subset of the provided patient data as to obtain a coherent patient sample for training their model. By identifying and omitting patients that appear considerably different from the remaining patients, they successfully lessened the effect of study-specific batch-effects. Another interesting approach has been proposed by team A Bavarian dream (as pointed out by the reviewer above). They used recalibration methods to adapt their model to the target study,* *which was* *used for final evaluation. In conclusion, we believe that the biggest improvements in risk prediction were not due to identifying new risk markers, but by choosing methods that account for sub-structures in the data. More research is needed to reliably detect such sub-structures and to overcome the problems they attend.*
